# Two Triacylglycerol Lipases Are Negative Regulators of Chilling Stress Tolerance in Arabidopsis

**DOI:** 10.3390/ijms23063380

**Published:** 2022-03-21

**Authors:** Ling Wang, Bilian Qian, Lei Zhao, Ming-Hua Liang, Xiangqiang Zhan, Jianhua Zhu

**Affiliations:** 1 School of Biotechnology, Jiangsu University of Science and Technology, Zhenjiang 212018, China; wanglingbear@163.com; 2 Department of Plant Science and Landscape Architecture, University of Maryland, College Park, MD 20742, USA; bilian.qian@outlook.com (B.Q.); zhaolei_tea@163.com (L.Z.); mhliangwing@163.com (M.-H.L.); 3 State Key Laboratory of Crop Stress Biology for Arid Areas, College of Horticulture, Northwest A&F University, Yangling, Xianyang 712100, China; zhanxq77@nwsuaf.edu.cn

**Keywords:** cold stress, triacylglycerol lipases, chilling stress tolerance, Arabidopsis

## Abstract

Cold stress is one of the abiotic stress conditions that severely limit plant growth and development and productivity. Triacylglycerol lipases are important metabolic enzymes for the catabolism of triacylglycerols and, therefore, play important roles in cellular activities including seed germination and early seedling establishment. However, whether they play a role in cold stress responses remains unknown. In this study, we characterized two Arabidopsis triacylglycerol lipases, MPL1 and LIP1 and defined their role in cold stress. The expression of *MPL1* and *LIP1* is reduced by cold stress, suggesting that they may be negative factors related to cold stress. Indeed, we found that loss-of-function of *MPL1* and *LIP1* resulted in increased cold tolerance and that the *mpl1lip1* double mutant displayed an additive effect on cold tolerance. We performed RNA-seq analysis to reveal the global effect of the *mpl1* and *lip1* mutations on gene expression under cold stress. The *mpl1* mutation had a small effect on gene expression under both under control and cold stress conditions whereas the *lip1* mutation caused a much stronger effect on gene expression under control and cold stress conditions. The *mpl1lip1* double mutant had a moderate effect on gene expression under control and cold stress conditions. Together, our results indicate that MPL1 and LIP1 triacylglycerol lipases are negative regulators of cold tolerance without any side effects on growth in Arabidopsis and that they might be ideal candidates for breeding cold-tolerant crops through genome editing technology.

## 1. Introduction

Cold stress significantly limits plant growth and development and causes substantial yield losses in crops worldwide. Cold stress can be experienced by plants as chilling stress and freezing stress. Chilling stress is critical for tropical and subtropical plants including tomato, soybean, sweet potato, and eggplant because they are not adapted to unpredicted sudden temperature drops (i.e., unforeseen frosts). Very little is known about the molecular nature of chilling stress responses in plants, whereas the molecular mechanisms of freezing stress tolerance in plants have been extensively studied. Cold temperature rapidly induces the expression of a small group of Apetala2 (AP2)/ethylene response factor (ERF)-type transcription factors termed dehydration responsive element (DRE)/C-repeat (CRT)-binding factors (CBFs/DREBs), which in turn control the expression of cold-responsive (*COR*) genes through binding to the DRE/CRT *cis*-element (consensus sequence of CCGAC) in the *COR* gene promoter regions [1,2,3]. The inducer of *CBF* expression 1 (ICE1), which is a basic helix–loop–helix transcription factor, functions as an upstream regulator of CBFs by binding to the MYC recognition sequences in the *CBF3* gene promoter and induces its expression under cold stress [4]. The ICE1 itself is subject to posttranslational modifications including phosphorylation, ubiquitination, and sumoylation under cold stress. A RING finger protein, HOS1, acts as an E3 ligase to mediate the polyubiquitination of ICE1 for degradation by the 26S protease complex under cold stress. Phosphorylation of ICE1 is controlled by multiple protein kinases. Open stomata 1 (OST1), a serine/threonine protein kinase involved in ABA signaling, phosphorylates ICE1 and suppresses the HOS1-mediated ICE1 degradation under cold stress [5]. Thus, OST1 functions as a positive regulator of the ICE1–CBF3 pathway for freezing tolerance. A recent study showed that two mitogen-activated protein kinases, MPK3 and MPK6, phosphorylate ICE1 and promote its degradation under cold stress to attenuate the ICE1–CBF3 pathway, while two calcium/calmodulin-regulated receptor-like kinases (CRLK1 and CRLK2) and MPK4 negatively regulate the kinase activities of MPK3 and MPK6 to allow the accumulation of ICE1 under cold stress [6]. It had been shown that SIZ1, a SUMO E3 ligase, facilitates sumoylation of ICE1 and that this posttranslational modification may activate and/or stabilize ICE1 to permit *CBF3* gene expression, leading to freezing tolerance [7].

Triacylglycerols (TAGs), three fatty acids esterified to glycerol, are major storage reserves of carbon and energy for oil seeds, pollens, and fruits, and they constitute up to 60% of the dry mass of oil seeds, while TAGs only make up a small percentage of the total lipids TAGs in vegetative tissues such as leaves and roots. TAG lipases de-esterify fatty acids from TAGs at each of the sn1, sn2, and sn3 positions. The active site of a TAG lipase consists of three amino acids (Ser, Asp or Glu, and His). TAG lipases can also hydrolyze diacylglycerols (DAGs) and other substrates including monoacylglycerol (MAGs), glycerol(phospho)lipids in position sn1, or sterol esters [8,9,10]. Therefore, TAGs are the major sources of fatty acids, which can be further metabolized through β-oxidation in peroxisomes to yield acetyl CoA, a key metabolite for energy production in mitochondrial respiration and for the synthesis of carbohydrates through the glyoxylate cycle and gluconeogenesis during seed germination and early seedling establishment. They are also critical for the normal growth and development of adult plants. DAGs can be used for the assembly of membrane lipids. TAG turnover by TAG lipases also accounts for the low accumulation of TAGs in vegetative tissues [11,12]. Characterization of a plastid TAG lipase from Arabidopsis revealed that TAG lipase is important in maintaining the structural integrity of chloroplasts, possibly by mobilizing the fatty acids of plastoglobular TAG [13]. It had been shown that the Arabidopsis MPL1 (MYZUS PERSICAE-INDUCED LIPASE1; encoded by *At5g14180*) has lipase activity and that it plays an important role against the green peach aphid [14]. The Arabidopsis LIP1 (encoded by *At2g15230*) has TAG lipase activity and *lip1* seedlings displayed no altered growth rates under normal growth conditions, but the *lip1* mutant seedlings accumulated more TAG than the wild type [15].

In this study, we aimed to determine the role of TAG lipases in cold stress responses through detailed characterization of the Arabidopsis *mpl1* and *lip1* single mutants and the double-mutant *mpl1lip1*. Our results indicate that MPL1 and LIP1 are partially redundant in cold stress responses and that both are negative regulators of chilling stress tolerance.

## 2. Results

### 2.1. Phylogenetic Analysis of MPL1 and LIP1, Expression Profiles of MPL1 and LIP1, and Generation of mpl1lip1 Double Mutant

**Figure 1:**
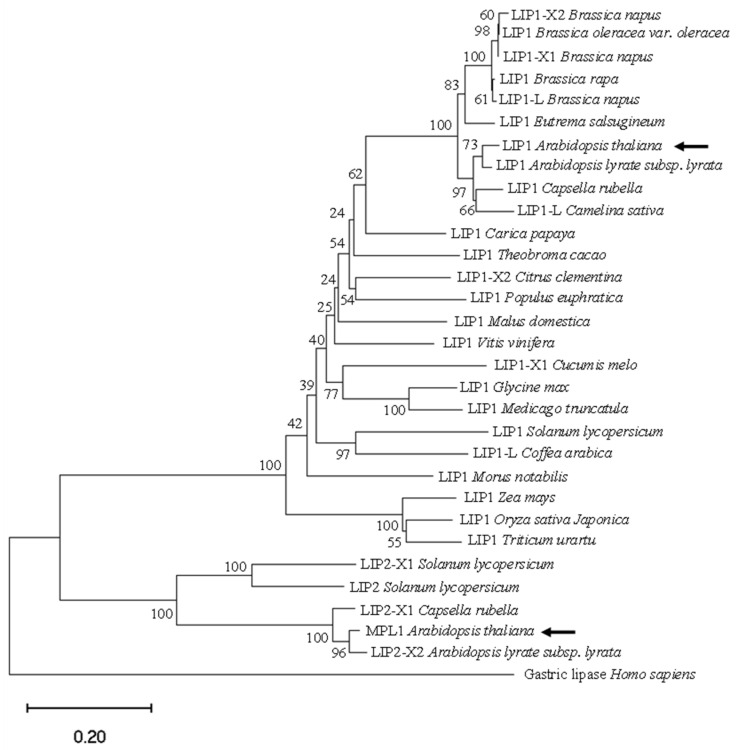
The evolutionary relationships of LIP1, MPL1, and their close homologs. The evolutionary tree was generated with MEGA11 using the neighbor-joining method with the JTT-matrix-based model. The percentage of replicate trees in which the associated taxa clustered together in the bootstrap test (1000 replicates) is shown next to the branches. Identities of the proteins shown in this tree are provided in the Materials and Methods section.

**Figure 2:**
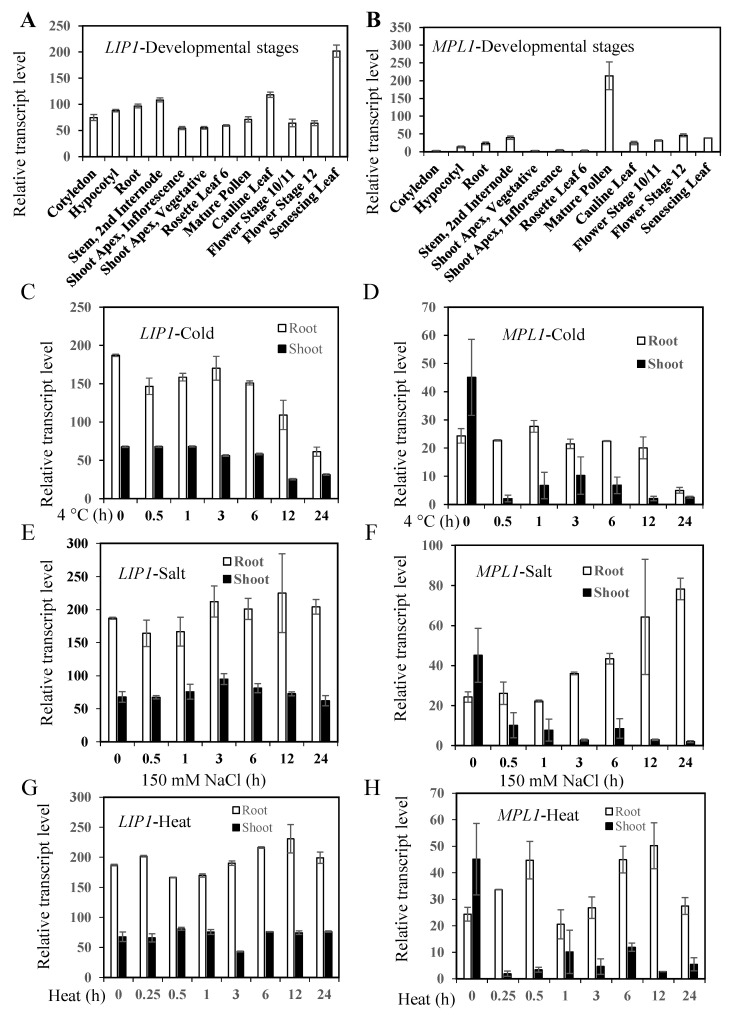
*LIP1* and *MPL1* display differential expression patterns in organs and developmental stages and under cold stress. (**A**,**B**) Expression profiles of *LIP1* (*At2g15230*) and *MPL1* (*At5g14180*) in different organs and developmental stages of wild-type plants as determined by microarrays [16]. (**C**,**D**) Expression profiles of *LIP1* and *MPL1* under cold stress as determined by microarrays with 13-day-old wild-type seedlings subjected to cold treatments [17]. (**E**,**F**) Expression profiles of *LIP1* and *MPL1* under salt stress as determined by microarrays with 13-day-old wild-type seedlings subjected to cold treatments [17]. (**G**,**H**) Expression profiles of *LIP1* and *MPL1* under salt stress as determined by microarrays with 16-day-old wild-type seedlings subjected to heat stress at 38 °C for 0, 0.25 (+0 h recovery at 25 °C), 0.5 (+0 h recovery at 25 °C), 1 (+0 h recovery at 25 °C), or 3 h (+3 h recovery at 25 °C = 6 h; +9 h recovery at 25 °C = 12 h; +21 h recovery at 25 °C = 24 h) [17]. Data are means ± sd (*n* = 3).

**Figure 3:**
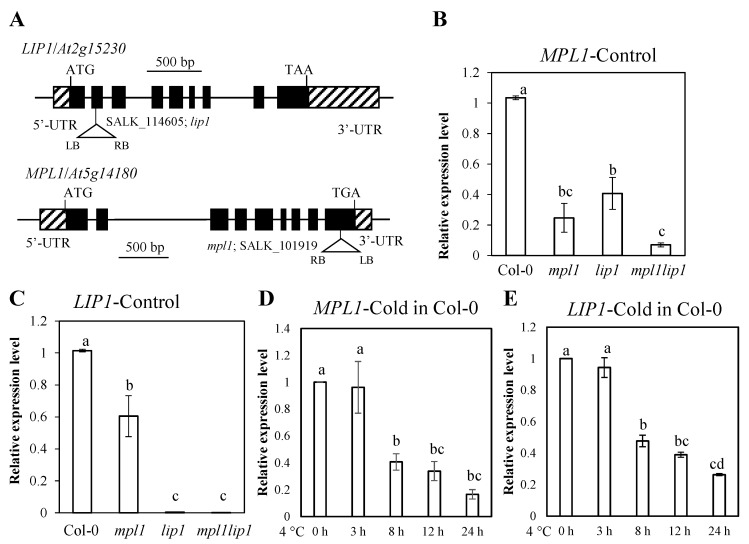
*MPL1* and *LIP1* expression level in Col-0, *mpl1* (*SALK_101919*), *lip1* (*SALK_114605*), and *mpl1lip1* plants under different conditions. (**A**) Gene structures of *MPL1* and *LIP1* with T-DNA insertions. *mpl1*: SALK_101919, *lip1*: SALK_114605. (**B**,**C**) Expression levels of *MPL1* and *LIP1* in two-week-old Col-0, *mpl1*, *lip1*, and *mpl1lip1* seedlings under control conditions. (**D**,**E**) Expression profiles of *MPL1* and *LIP1* in two-week-old Col-0 seedlings subjected to cold stress (4 °C). Data are means ± sd (*n* = 4). One-way ANOVA (Tukey test) was performed, and significant difference is indicated by different lowercase letters (*p* < 0.05).

To assess the function of triacylglycerol lipases in cold stress responses, we examined expression profiles of two triacylglycerol lipases from *Arabidopsis thaliana*, *MPL1* and *LIP1*. MPL1 and LIP1 share high sequence homologies with triacylglycerol lipases from other plant species (Figure 1). *LIP1* is expressed in all plant tissues and reaches peak expression level in senescing leaves (Figure 2A), while *MPL1* is expressed at relatively lower levels than *MPL1* and its peak expression is found in the mature pollens (Figure 2B). The expression of *LIP1* is downregulated by cold stress in the root tissues (Figure 2C) or in whole seedlings (Figure 3E). The expression of *MPL1* is downregulated by cold stress in the shoot tissues (Figure 2D) or in whole seedlings (Figure 3D). These results suggest that MPL1 and LIP1 might act as negative regulators of cold stress. Essentially *LIP1* is not responsive to salt stress or heat stress (Figure 2E,G). *MPL1* is moderately upregulated in roots or downregulated in shoots under salt stress (Figure 2F). *MPL1* is also downregulated in shoots under heat stress (Figure 2H). These results suggest that MPL1 may play a role in salt stress or heat stress.

We obtained two T-DNA insertional mutants for *MPL1* (SALK_101919; *mpl1*) and *LIP1* (SALK_114605; *lip1*) and identified homozygous mutant plants through genotyping (Figure 3A). The expression of *MPL1* was significantly reduced in the *mpl1* seedlings, and the expression of *LIP1* was almost undetectable in the *lip1* seedlings (Figure 3B,C). The expression of *MPL1* in the *lip1* seedlings and the expression of the *LIP1* in the *mpl1* seedlings were significantly reduced, suggesting that there is a feedback regulation of *MPL1* by the *lip1* mutation and there is a feedback regulation of *LIP1* by the *mpl1* mutation (Figure 3B,C). To overcome potential functional redundance of *MPL1* and *LIP1*, we generated the *mpl1lip1* double mutant by crossing the *mpl1* and *lip1* single-mutant plants. The expression of the *MPL1* and *LIP1* was significantly low in the *mpl1lip1* double-mutant plants (Figure 3B,C).

### 2.2. The mpl1, lip1, and mpl1lip1 Plants Display Increased Chilling Stress Tolerance at Seed Germination and Post-Germination Seedling Development Stages

**Figure 4:**
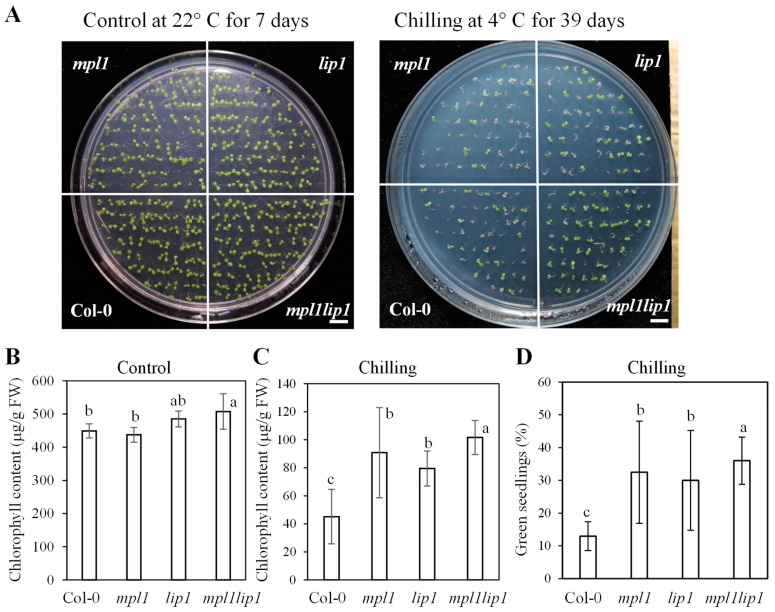
Chilling phenotype of *mpl1*, *lip1*, and *mpl1lip1* plants. (**A**–**D**) Seeds of Col-0, *mpl1*, *lip1*, and *mpl1lip1* were sown on MS medium plates and immediately incubated in growth chambers at 22 °C for 7 days (Control) or at 4 °C (Chilling) for 39 days under a long-day photoperiod (16 h day and 8 h night; light intensity ~80 μmol/m^2^/s). There were 50–80 seedlings for each genotype per MS plate. (**A**) Morphology of Col-0, *mpl1*, *lip1*, and *mpl1lip1* plants under control and chilling conditions. Bar = 1 cm. (**B**) Chlorophyll content of the plants shown in (**A**) under control conditions. (**C**) Chlorophyll content of the plants shown in (**A**) under chilling stress for 39 days. (**D**) Percentage of green seedlings of Col-0, *mpl1*, *lip1*, and *mpl1lip1* under chilling stress for 39 days. Data are means ± sd (*n* = 3 in (**B**), ≥6 in (**C**,**D**) (*n* indicates the number of MS medium plates)). One-way ANOVA (Tukey test) was performed, and significant difference is indicated by different lowercase letters (0.007 < *p* < 0.01).

Frost in late fall or early spring can be a limiting factor for seed germination of temperate plant species. We simulated the frost conditions by incubating MS agar plates with freshly sown seeds at chilling temperature in a growth chamber. It took about several weeks for the seeds to germinate, and approximately two additional weeks for the seedlings with fully expanded cotyledons and the first leaf to emerge. Under control conditions, there were no morphological differences among the *mpl1*, *lip1*, *mpl1lip1,* and Col-0 seedlings and the *mpl1lip1* seedlings accumulated slightly more chlorophyll than the Col-0 seedlings (Figure 4A,B). Under chilling stress, the *mpl1* and *lip1* plants were moderately chilling tolerant as indicated by more green seedlings and increased accumulation of chlorophyll (Figure 4 A,C,D). The *mpl1lip1* plants showed additive effect of the *mpl1* and *lip1* mutations—that is the *mpl1lip1* plants were chilling tolerant with proportionally more green seedlings and increased accumulation of chlorophylls than the *mpl1* and *lip1* single mutants under chilling stress (Figure 4 A,C,D). These results suggest that MPL1 and LIP1 are negative regulators of chilling stress tolerance.

### 2.3. The mpl1, lip1 and mpl1lip1 Plants Are Chilling Tolerant When Grown in the Dark

**Figure 5:**
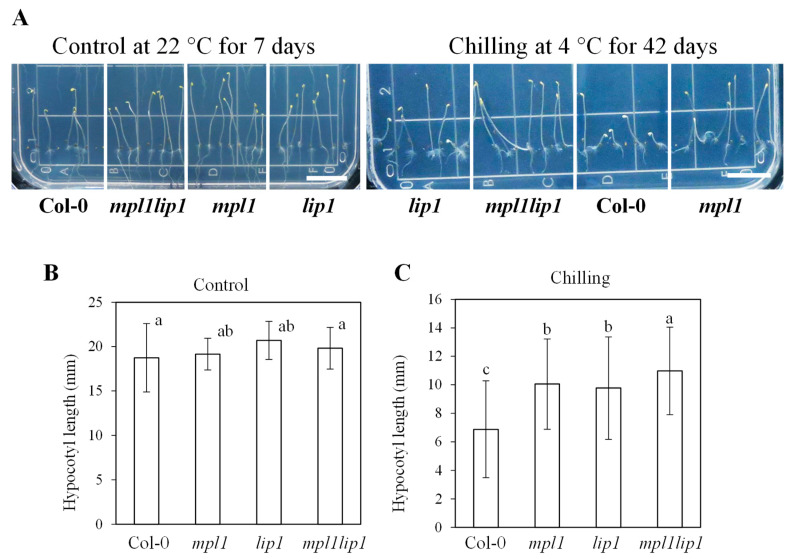
Hypocotyl elongation of the *mpl1*, *lip1*, and *mpl1lip1* seedlings under chilling stress. (**A**–**C**) Seeds of Col-0, *mpl1*, *lip1*, and *mpl1lip1* were sown in two rows on vertical MS medium plates and immediately incubated in growth chambers while kept in darkness at 22 °C for 7 days (Control) or at 4 °C (Chilling) for 42 days. There are 14–20 seeds for each genotype per MS medium plate. (**A**) Morphology of Col-0, *mpl1*, *lip1*, and *mpl1lip1* under control and chilling conditions. (**B**) Hypocotyl elongation of Col-0, *mpl1*, *lip1*, and *mpl1lip1* under control conditions for 7 days. (**C**) Hypocotyl elongation of Col-0, *mpl1*, *lip1*, and *mpl1lip1* under chilling stress for 42 days. Data are means ± sd (*n* = 4 in (**B**) 12 in (**C**) (*n* indicates the number of MS medium plates)). One-way ANOVA (Tukey test) was performed, and significant difference is indicated by different lowercase letters (*p* < 0.03).

To determine whether the increased chilling stress tolerance in the *mpl1*, *lip1,* and *mpl1lip1* plants depends on the developmental stages, we examined the response of the *mpl1*, *lip1,* and *mpl1lip1* seedlings to chilling temperature in the dark after their seeds had germinated. We measured the hypocotyl elongation as an indicator of chilling stress responses. Essentially there were no differences in hypocotyl elongation among Col-0, *mpl1*, *lip1,* and *mpl1lip1* seedlings under control conditions (Figure 5A,B). Under chilling stress, the *mpl1*, *lip1,* and *mpl1lip1* seedlings developed significantly longer hypocotyls than the Col-0 seedlings, indicating that they were chilling tolerant (Figure 5A,C).

### 2.4. The mpl1, lip1, and mpl1lip1 Mutations Do Not Alter the Responses to Freezing Temperatures or Heat Stress

**Figure 6:**
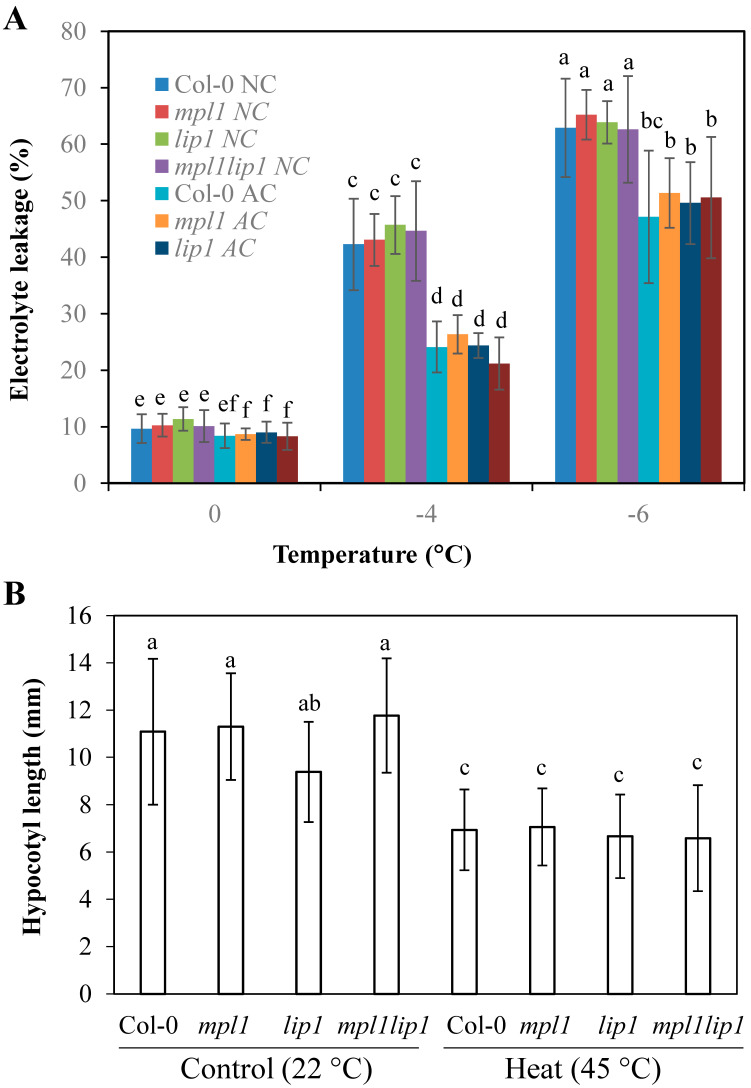
Responses of the *mpl1*, *lip1*, and *mpl1lip1* plants to freezing and heat stresses. (**A**) Electrolyte leakage indictive of damage of biological membranes caused by freezing temperatures. NC, non-cold acclimated; AC, cold acclimated. (**B**) Hypocotyl elongation of *mpl1*, *lip1*, and *mpl1lip1* in response to heat stress. Seeds were sown in two rows on vertical MS medium plates and incubated at 22 °C for 36 h in darkness to ensure uniform seed germination and initial hypocotyl elongation. There were 14–20 seeds for each genotype per MS medium plate. The germinated seedlings were treated at 45 °C for 0 or 1 h and allowed to grow in darkness in a growth room at 22 °C for an additional 3 days. Data are means ± sd (*n* = 18 in (**A**) (*n* = number of detached leaves), 4 (Control), 9 (Heat) in (**B**) (*n* = number of MS medium plates)). One-way ANOVA (Tukey test) was performed, and significant difference was indicated by different lowercase letters (*p* < 0.05).

We speculate that the *mpl1*, *lip1,* and *mpl1lip1* mutations may cause additional phenotypic alterations to other abiotic stress conditions. In addition to chilling stress, freezing stress is the second component of the cold stress. We examined the responses of the *mpl1*, *lip1,* and *mpl1lip1* plants to freezing temperatures with detached rosette leaves from soil-grown plants. Without cold acclimation, the *mpl1*, *lip1,* and *mpl1lip1* plants released similar amounts of electrolytes from damaged biological membranes caused by freezing temperatures as the Col-0 plants (Figure 6A). After cold acclimation at 4 °C for one week, the *mpl1*, *lip1,* and *mpl1lip1* plants released similar amounts of electrolytes from damaged biological membranes caused by freezing temperatures to the Col-0 plants (Figure 6A). These results indicate that the *mpl1*, *lip1,* and *mpl1lip1* mutations do not alter the responses to freezing temperatures.

We subsequently examined the responses of the *mpl1*, *lip1,* and *mpl1lip1* plants to another temperature extreme, heat. We used hypocotyl elongation of heat-treated seedlings that grew in the dark as an indicator for heat stress responses. All plants tested displayed similar length of hypocotyls under control conditions, and they had similarly reduced length of hypocotyls by one-hour treatment at 45 °C (Figure 6B). These data indicate that the *mpl1*, *lip1,* and *mpl1lip1* mutations do not alter the responses to heat stress.

### 2.5. The mpl1, lip1, and mpl1lip1 Mutations Alter Gene Expression under Chilling Stress

**Figure 7:**
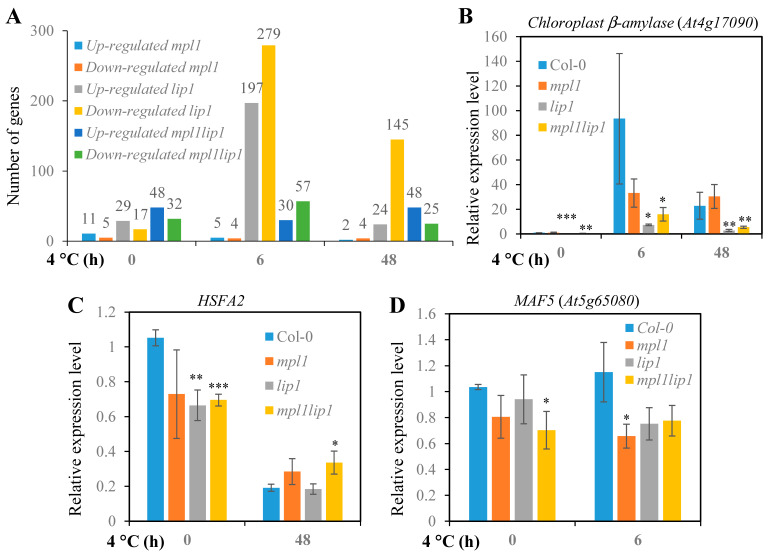
Deferentially expressed genes in the *mpl1*, *lip1,* and *mpl1lip1* plants determined by RNA-seq analysis. The RNA-seq and qRT-PCR experiments were performed with two-week-old seedlings subjected to cold stress at 4 °C for 0, 6, or 48 h. (**A**) Differentially expressed genes in the *mpl1*, *lip1,* and *mpl1lip1* plants determined by RNA-seq analysis. (**B**–**D**) Validation of the RNA-seq results by qRT-PCR analysis. Values represent means ± se (*n* = 4 (n indicates number of experiments and there were 4 biological replicates in each experiment)). Significant differences in mean values are indicted by asterisk(s) determined by Student’s t-tests (* *p* < 0.05; ** *p* < 0.01; *** *p* < 0.001).

We performed RNA-seq experiments to determine whether the *mpl1*, *lip1,* and *mpl1lip1* mutations affect gene expression (Supplemental Table S1). Data analysis of the RNA-seq results revealed that the *mpl1* mutation had a modest effect on gene expression under both control and chilling stress conditions (Figure 7A and Supplemental Tables S2–S4). Our RNA-seq analysis also revealed that the *lip1* mutation had a relatively strong effect on the gene expression under control and chilling stress conditions (Figure 7A and Supplemental Tables S5–S7). Compared to the *mpl1* and *lip1* single mutations, the *mpl1lip1* double mutations had a moderate effect on gene expression under control and chilling stress conditions (Figure 7A and Supplemental Tables S8–S10). The RNA-seq analysis appears to be reliable because reductions in the expression of *MPL1* and *LIP1* were detected in the relevant mutant background (Supplemental Tables S3–S10). We subsequently validated the RNA-seq results by qRT-PCR analysis with three randomly selected genes (Figure 7B–D).

The differentially expressed genes in the *mpl1*, *lip1,* and *mpl1lip1* plants encode proteins with diverse functions. In the *mpl1* plants, 6 h of cold stress led to altered expression of nine genes and these genes encode proteins for cellular metabolism, protein synthesis and turnover, and responses to abiotic stresses (Supplemental Table S3. Exposure to cold stress for 48 h in the *mpl1* plants resulted in altered expression of six genes, and these genes encode proteins for protein turnover, cellular metabolism, and plant development (Supplemental Table S4). It was apparent that short-term cold treatment (6 h) causes changed in the expression of more genes than the long-term cold treatment did (48 h) (Supplemental Tables S2 and S3). Cold treatment for 6 h altered the expression of 476 genes in the *lip1* plants; a large portion of them encode proteins for cellular metabolism including lipid metabolism, and some of the genes encode components of the spliceosome for RNA processing, transcription factors for gene regulation, and enzymes for ROS detoxification (Supplemental Table S6). Cold treatment for 48 h in the *lip1* plants had less effect on gene expression, and 169 genes displayed altered expression levels and these genes encode proteins with similar functions to those encoded by differentially expressed genes 6 h after cold treatment (Supplemental Table S7). Eighty-seven genes were differentially expressed in the *mpl1lip1* plants after 6 h exposure to cold stress, and the expression of 73 genes was altered in the *mpl1lip1* plants by cold treatment for 48 h (Supplemental Tables S9 and S10). The differentially expressed genes in the *mpl1lip1* plants encode proteins involved in cellular metabolism, gene expression regulation, protein turnover, ROS detoxification and stress responses.

## 3. Discussion

Lipid biosynthesis and catabolism are critical for plant growth and development processes such as seed germination, seedling establishment, and seed development [18]. Triacylglycerols are important for membrane lipid breakdown, fatty acid b-oxidation, and plant survival under prolonged darkness conditions [19]. Triacylglycerol lipases carry out the catabolic reactions of triacylglycerol breakdown. It was shown that triacylglycerol lipases are important for seed germination, early seedling establishment, and normal growth and development of mature plants; however, their role in cold stress responses is not clear. In this study, we characterized two triacylglycerol lipases, MPL1 and LIP1, and defined their function in cold stress. The expression of both *MPL1* and *LIP1* was downregulated by cold stress, suggesting that they may function as negative regulators for cold stress. We suspect that there might be high level of functional redundancy between MPL1 and LIP1 for cold stress. To overcome this potential functional redundancy, we created the double mutant of *mpl1lip1*. To our surprise, the single-mutant plants of both *mpl1* and *lip1* displayed detectable phenotypic changes under cold stress. Both the *mpl1* and *lip1* plants were tolerant to chilling stress at multiple developmental stages. The *mpl1lip1* plants were moderately more tolerant to the chilling stress conditions than the individual single-mutant plants, indicating that there is some level of additive effect of the *mpl1* and *lip1* mutations. Therefore, MPL1 and LIP1 display partial functional redundancy under chilling stress. We observed that the *mpl1*, *lip1,* and *mpl1lip1* plants did not have altered responses to freezing stress and heat stress, suggesting that MPL1 and LIP1 have a specific function in chilling stress conditions. This observation is also consistent with their unique expression patterns under abiotic stresses. Because the *mpl1lip1* double mutant does not show any detectable growth defects under both normal and cold stress conditions, the *MPL1* and *LIP1* might be ideal loci for breeding chilling-tolerant crops.

Mutations of *MPL1* and *LIP1* led to altered expression of genes under normal and cold stress conditions. The functions of those genes with differential expression levels in the *mpl1*, *lip1,* and *mpl1lip1* plants under normal growth conditions were not apparent because these mutant plants did not show detectable phenotypic changes under normal conditions. Under cold stress conditions, there were more differentially expressed genes in the mutant plants after 6 h of cold treatment than in the plants exposed to a 48 h cold stress, presumably representing the changes in the expression of the early response genes. The differentially expressed genes under cold stress encode proteins with diverse functions, including metabolic enzymes for lipids, carbohydrates, and proteins; regulators of gene expression such as transcription factors; and components of the spliceosome. Collectively, the altered expression of these genes contributes to the increased chilling stress tolerance observed in the *mpl1*, *lip1,* and *mpl1lip1* plants.

It has been shown that accumulation of lipid intermediates such as DAGs, free fatty acids, and membrane phospholipids is associated with premature cell death in growing leaves and floral organs [20]. Therefore, TAG synthesis or disruption of TAG breakdown can protect against free fatty acid–induced cell death in plant vegetative tissues. This, probably, at least in part, explains why the *mpl1*, *lip1,* and *mpl1lip1* plants are tolerant to chilling stress. Furthermore, abiotic stress conditions including cold stress can cause altered membrane lipid composition through lipid remodeling to enhance the maintenance of membrane fluidity, stability, and integrity [21,22]. For example, heat stress induces the degradation of monogalactosyldiacylglycerol (MGDG) and chlorophyll in chloroplasts, leading to the accumulation of toxic lipid intermediates including DAGs, free fatty acids, and phytyl, which may damage plant tissues [23]. Under chilling stress conditions, the disruptions in TAG breakdown in the *mpl1*, *lip1,* and *mpl1lip1* plants would have a profound impact on lipid metabolism including accumulation of TAGs and altered biological membrane lipid compositions. The accumulated TAGs could sequester the cold-induced toxic lipid intermediates to avoid cell death, thereby achieving improved chilling stress tolerance.

## 4. Materials and Methods

### 4.1. Plant Materials and Growth Conditions

*Arabidopsis thaliana* seedlings of Col-0, *mpl1* (stock number SALK_101919), *lip1* (stock number SALK_114605), and *mpl1lip1* (created from SALK_101919 and SALK_114605) on Murashige and Skoog (MS) medium agar plates (1× MS salts, 2% sucrose, and 0.6% or 1.2% agar, pH 5.7) were routinely grown under cool, white light (~120 μmol m^−2^ s^−1^) at 22 ± 1 °C with a 16-h-light/8-h-dark photoperiod. Soil-grown plants were kept under cool, white light (~100 μmol m^−2^ s^−1^) with a 16-h-light/8-h-dark photoperiod at 22 ± 1 °C and with a 1:1 ratio of Metro Mix 360 and LC1 potting soil (Sun Gro Horticulture, Agawam, MA, USA).

### 4.2. Chilling and Freezing Tolerance Assays

For chilling tolerance assay to examine the ability of seed germination and seedling development under light, seeds of the relevant genotypes were sown side by side on MS agar plates containing 0.6% agar. These agar plates were kept horizontally at 4 or 22 °C in growth chambers with a 16-h-light/8-h-dark photoperiod for the desired time.

For chilling tolerance assay to examine hypocotyl elongation in the dark, seeds of the relevant genotypes were sown side by side on MS agar plates containing 1.2% agar. These agar plates were then wrapped with aluminum foil and kept vertically at 4 or 22 °C in growth chambers for the desired time. Hypocotyl elongation at 4 °C relative to that at 22 °C was used as an indicator of sensitivity to chilling stress.

To evaluate freezing tolerance using electrolyte leakage assays, 3-week-old Col-0, *mpl1*, *lip1,* and *mpl1lip1* plants were grown in soil at room temperature or at 4 °C under a 16-h-light/8-h-dark photoperiod for one week. Fully developed rosette leaves were used for electrolyte leakage measurements as described in [24,25,26].

### 4.3. Determination of Chlorophyll Content

Chlorophyll was determined as described in [27] with minor modifications. Seedlings were frozen in liquid nitrogen and ground to fine powder. Chlorophyll was extracted by incubating ground tissues in 80% acetone overnight at 4 °C in darkness and with continuous shaking. The contents of chlorophyll a and b were calculated as 7.49 A_664.9_ + 20.3 A_648.2_.

### 4.4. RNA-seq Analysis and qRT-PCR Analysis

Fourteen-day-old Col-0, *mpl1*, *lip1,* and *mpl1lip1* seedlings grown on MS medium (1× MS salts, 2% sucrose, 0.6% agar, pH 5.7) were treated at 4 °C for 0, 6, or 48 h and were used for total RNA extraction. Total RNA was isolated with the Universal Plant Total RNA Extraction Kit (BioTeke, Beijing, China) and treated with a TURBO DNA-free™ Kit (Thermo Fisher Scientific, Waltham, MA, USA) to remove any genomic DNA contaminants. mRNA-seq libraries were constructed following the standard Illumina protocols. There were three biological replicates per genotype. Illumina sequencing was performed in the Shanghai Center for Plant Stress Biology with an Illumina HiSeq 2500 system. 

For each sample, RNA-seq raw reads were trimmed using Trimmomatic v0.32 [28] and PRINSEQ v0.20.4 [29]. Trimmomatic was used to remove the potential Illumina adapter contamination and conduct reads while trimming and clipping the low-quality bases. PRINSEQ was subsequently employed to mainly remove low-complexity reads. The remained reads were then aligned to the *Arabidopsis thaliana* genome sequence and the reference annotated genes (TAIR10) using the TopHat v2.0.13 program [30] with customized parameters specific for our RNA-seq libraries from the *Arabidopsis thaliana* plants (—read-edit-dist 3—read-realign-edit-dist 0—b2-very-sensitive -r 150—mate-std-dev 200 -a 6 -i 8 -I 10,000—min-segment-intron 8—max-segment-intron 10,000—microexon-search).

According to RNA-seq mapped reads and the reference annotated transcripts, transcriptomes were reconstructed for each sample using Cufflinks v2.2.1 [31]. Given the variable efficiency of mRNA enrichments and rRNA depletion kits in samples, these transcripts were masked in transcriptome constructions to improve the overall robustness of transcript abundance estimates. To obtain a high confidence of transcriptomes, the novel constructed transcript was filtered out when the abundance was lower than 20% (default is 10%) of the most abundant isoform for each gene. Then, all the constructed transcriptomes were merged with the reference annotated transcripts using Cuffmerge [31] to yield comprehensive re-annotated gene transcripts including known and novel annotated transcripts in our RNA-seq samples. Subsequently, significantly differentially expressed genes were predicted using Cuffdiff [31] between the controls and tested samples, using a twofold-change and multiple test *p*-value < 0.05.

For qRT-PCR analysis, 5 µg of total RNA was used to synthesize the first-strand cDNA with the Maxima First-Strand cDNA synthesis kit ( Thermo Fisher Scientific, Waltham, MA, USA) as described [32]. Each experiment had four biological replicates (two technical replicates for each biological replicate), and each experiment was repeated at least three times. The comparative cycle threshold method was applied, and *UBQ5* was used as a reference gene. The gene-specific primers are listed in the Supplemental Table S11.

### 4.5. Phylogenetic Tree Construction

Close homologs of MPL1 and LIP1 from other plant species were identified through NCBI BLASTP searches. We also selected one lipase from the human genome. The full-length protein sequences of the MPL1, LIP1, and their homologs were aligned in MEGA11 with MUSCLE, and the phylogenetic tree was constructed with the neighbor-joining method (Jones–Taylor–Thornton model; 1000 bootstrap replicates) [33]. The identities of the proteins in the phylogenetic tree are as follows: LIP1-X2 *Brassica napus* (accession number XP_013705879.1), LIP1 *Brassica oleracea* var. *oleracea* (XP_013593569.1), LIP1-X1 *Brassica napus* (XP_013705814.1), LIP1 *Brassica rapa* (XP_009102330.1), LIP1-L *Brassica napus* (XP_013653884.1), LIP1 *Eutrema salsugineum* (XP_006409584.1), LIP1 *Arabidopsis thaliana* (NP_179126.2), LIP1 *Arabidopsis lyrata* subsp. *lyrata* (XP_002883884.1), LIP1 *Capsella rubella* (XP_006296713.1), LIP1-L *Camelina sativa* (XP_010468612.1), LIP1 *Carica papaya* (XP_021892391.1), LIP1 *Theobroma cacao* (XP_017971739.1), LIP1-X2 *Citrus clementina* (XP_006422918.1), LIP1 *Populus euphratica* (XP_011001191.1), LIP1 *Malus domestica* (XP_008338234.1), LIP1 *Vitis vinifera* (XP_019074684.1), LIP1-X1 *Cucumis melo* (XP_008454984.1), LIP1 *Glycine max* (XP_003536618.1), LIP1 *Medicago truncatula* (XP_013470044.1), LIP1 *Solanum lycopersicum* (XP_004250654.1), LIP1-L *Coffea arabica* (XP_027112568.1), LIP1 *Morus notabilis* (XP_024021284.1), LIP1 *Zea mays* (PWZ15208.1), LIP1 *Oryza sativa Japonica Group* (XP_015651228.1), LIP1 *Triticum urartu* (EMS51240.1), LIP2-X1 *Solanum lycopersicum* (XP_004233301.1), LIP2 *Solanum lycopersicum* (XP_004234001.1), LIP- X1 *Capsella rubella* (XP_006286780.1), MPL1 *Arabidopsis thaliana* (NP_568295.2), LIP2-X2 *Arabidopsis lyrata* subsp. *lyrata* (XP_020876076.1), and Gastric lipase *Homo sapiens* (P07098.1).

### 4.6. Statistical Analysis

One-way analysis of variance (ANOVA; Tukey test) was performed, and significant difference is indicated by different lowercase letters (*p* < 0.05). When applicable, significant differences in mean values are indicted by asterisk(s) determined by Student’s *t*-tests (* *p* < 0.05; ** *p* < 0.01; *** *p* < 0.001).

## Data Availability

Not applicable.

## Supplementary Materials

The following supporting information can be downloaded at: https://www.mdpi.com/article/10.3390/ijms23063380/s1.
